# Mortality in clostridioides difficile infection among patients hospitalized at the university clinical hospital in Wroclaw, Poland – a 3-year observational study

**DOI:** 10.1186/s12879-024-09495-7

**Published:** 2024-06-23

**Authors:** Jarosław Drobnik, Piotr Pobrotyn, Mária Belovičová, Katarzyna Madziarska, Małgorzata Trocha, Mateusz Baran

**Affiliations:** 1 Department of Family Medicine, Wroclaw Medical University, Wrocław, Poland; 2 PULSANTIS Specialist and Rehabilitation Clinic Ltd, Ostrowskiego 3, Wrocław, 53-238 Poland; 3 Faculty of Public Health Studies, Department of Preventive and Clinical Medicine, Slovak Medical University, Bratislava, Slovakia; 4 Internal Clinic for Liver Disease Diagnosis and Treatment, Bardejov Spa, Bardejov, Slovakia; 5 Slovak Society of Practical Obesitology, Bardejov, Slovakia; 6 Clinical Department of Diabetology and Internal Diseases, Wroclaw Medical University, Wroclaw, Poland; 7 Individual Specialist Medical Practice, Wroclaw, Poland

**Keywords:** Clostridioides difficile infection, Mortality risk, Malnutrition, Comorbidities, Hospitalization

## Abstract

**Background:**

In the last two decades, a significant increase in the number of Clostridioides difficile infection (CDI) cases has been observed. It is understandable to attempt to determine the factors that can predict the severity of the course of the infection and identify patients at risk of death. This study aimed to analyze the factors affecting the incidence and mortality of CDI in inpatient treatment at the University Clinical Hospital in Wrocław in 2016–2018.

**Methods:**

Statistical analysis of data obtained from patients’ medical records was performed. Only patients with symptoms of infection and infection confirmed by laboratory tests were enrolled in the study. When analyzing the number of deaths, only adult patients who died in hospital wards were included. The quantitative data including laboratory tests, used antibiotics and Nutritional Risk Screening (NRS) were assessed. Also, the qualitative data such as sex, year of hospitalization, occurrence of diarrhoea on admission to the hospital, presence of additional diseases, as wee ad the use of antibacterial drugs or proton pump blockers and ranitidine during hospitalization were analyzed.

**Results:**

A total of 319 adult CDI patients (178 women and 141 men) were enrolled of which 80 people died (50 women and 30 men). The mean age of the patients was 72.08 ± 16.74 years. Over the entire period studied, the morbidity was 174 cases per 100,000 hospitalizations while mortality was 25.08%. The group of deceased patients was characterized by: older age (by 9.24 years), longer duration of hospitalization (by 10 days), reduced albumin levels (Rho = -0.235, *p* < 0.001), higher urea levels, use of more antibiotics, higher risk of malnutrition in NRS (Rho = 0.219, *p* < 0.001), higher incidence of sepsis, heart failure, stroke, hypothyroidism. Pneumonia was diagnosed twice as often. It was also shown that deceased patients were significantly more likely to take penicillin and fluoroquinolones.

**Conclusions:**

In this study, the morbidity was lower, but mortality was higher compared to similar hospitals in Poland. CDI patients were characterized by older age, multimorbidity, extended hospitalization, and the use of broad-spectrum antibiotics. Risk factors for death included advanced age, prolonged hospital stays, lower albumin, higher urea, malnutrition, and comorbidities like heart failure, stroke, pneumonia, sepsis, and hypothyroidism. Increased antibiotic use, particularly penicillin and fluoroquinolones, was associated with a higher mortality risk.

## Background

Clostridioides difficile infection (CDI) is the most common cause of post-antibiotic diarrhea worldwide [1]. The condition of Clostridioides difficile infection (CDI) is characterized by diarrhea, and diagnosis typically requires the presence of diarrhea in conjunction with at least one of the following criteria, as outlined by IDSA/SHEA [2] or ESCMID [3] guidelines: (1) detection of toxin A and/or B in the stool or detection of the bacterial strain by another method, (2) identification of pseudomembranous enteritis during endoscopy or surgery, or (3) confirmation of pseudomembranous enteritis on histopathology [4].

CDI is a severe disease with a high mortality rate [5–7]. Its course is highly variable, from mildly symptomatic cases, requiring only discontinuation of the current antibiotic therapy, to complicated forms, including septic shock. Therefore, it is understandable to attempt to determine the factors that can predict the severity of the course of the infection and identify patients at risk of death.

In the last two decades, a significant increase in the CDI cases has been observed [8]. In Poland in 2016, the incidence was 22.7 per 100,000 inhabitants, 8716 infections were detected, and 7430 (85.2%) patients were hospitalized. In 2017–2020, these parameters increased: incidence ranged from 26.4 to 30.4/100,000 population, the number of infections detected from 10,139 to 11,667, and the number of hospitalized patients 7393–10,273 (85.7–88.1%). In 2021, the incidence increased even higher to 55.5 cases per 100,000 population, 21,174 infections were detected and 15,762 (74.4%) people were hospitalized [9–12].

Mortality in CDI is high and it ranged from 9 to 38%, depending on the study analyzed [13, 14]. The Polish study conducted in the years 2008–2014 at the University Clinical Hospital in Krakow showed that the exact mortality rate was 17.34% [15]. While these findings provide valuable insights, it is important to acknowledge the need for further research to better understand the factors influencing mortality in CDI.

Therefore, this study aimed to analyze the factors affecting the incidence and mortality of CDI in inpatient treatment at the University Clinical Hospital in Wrocław in 2016–2018 and to determine clinical data characterizing a group of deceased patients with CDI.

## Methods

### Sample and settings

The study analyzed the cases of all adult patients diagnosed with CDI among those hospitalized at the Jan Mikulicz-Radecki University Clinical Hospital in Wroclaw in 2016–2018. The diagnosis of CDI infection among patients at the University Clinical Hospital in Wrocław was based on a combination of diagnostic tests. Specifically, we utilized glutamate dehydrogenase (GDH) detection in conjunction with stool testing for toxins A and B using latex agglutination tests (enzyme-linked immunosorbent assay - ELISA). Additionally, nucleic acid amplification tests (NAAT) were employed for the detection of CDI [16, 17]. These diagnostic methods enabled accurate identification of CDI cases within our patient cohort, providing a robust foundation for our subsequent analyses of risk factors and outcomes associated with this infection.

Statistical analysis of data obtained from patients’ medical records was performed. Only patients with symptoms of infection and infection confirmed by laboratory tests were enrolled in the study. When analyzing the number of deaths, only adult patients who died in hospital wards were included. When determining the incidence, the number of cases (rounded to the whole) per 100,000 hospitalizations is given. Overall hospital mortality was calculated using the hospital database, from which the number of deaths and hospitalizations in individual wards and in individual years was obtained.

### Medical records

The following quantitative data were evaluated: age, length of hospitalization, laboratory results: erythrocyte sedimentation rate, C-reactive protein, number of leukocytes, erythrocytes, thrombocytes in blood, haematocrit, haemoglobin, sodium, potassium, magnesium, calcium, total protein, albumin, creatinine, urea, number of antibiotics (chemotherapeutics) used by patients during hospitalization, the number of points obtained in the Nutritional Risk Screening (NRS 2002), as an assessment of the risk of malnutrition in patients. NRS 2002 was used to evaluate the nutritional status of patients. It is a screening test used to assess health risks related to nutritional status. The NRS evaluates: body mass index (BMI), reduced food intake over the past week, the amount of unintentional weight loss expressed as a percentage of body weight, the presence of additional medical conditions, the patient’s age; patients over 70 years old receive an additional point on the scale. The scale can be scored from 0 to 7 points. A score of 3 points or more indicates a higher risk of malnutrition-related complications and indicates the need to include nutritional treatment. In patients with a score of less than 3 points, the scale should be repeated after 7 days of hospitalization [18].

If a patient had several tests of the same type performed, the earliest result was taken into account in order to collect data with prognostic value for the risk of death and the severity of the disease course. This limited the potential impact of the applied therapy on the results of laboratory tests. The following qualitative data were analysed: sex, year of hospitalization, occurrence of diarrhoea on admission to the hospital, presence of additional diseases: urinary tract infections, pneumonia, sepsis, use of antibacterial drugs (penicillins, cephalosporins, carbapenems, fluoroquinolones, aminoglycosides, macrolides, sulfamethoxazole and trimethoprim, colistin) and proton pump blockers and ranitidine during hospitalization.

Differences in the risk of death of patients, depending on the additional conditions they had, were analyzed. The following comorbidities were included: stroke, dementia, hypertension, ischemic heart disease, myocardial infarction, heart failure, atrial fibrillation, diabetes, chronic or acute kidney damage, inflammatory bowel disease (Crohn’s disease or ulcerative colitis), pressure ulcer or chronic ulceration, surgery during hospitalization, hypothyroidism, anaemia, active oncological process.

### Statistical analysis

All statistical analyses were performed using R software [19]. Relationships between quantitative and dichotomous variables were verified using the Student’s t-test and the Mann-Whitney test (when the distributions of the variables deviated significantly from the normal distribution). Relationships between qualitative variables were verified using the chi-square test (along with a post-hoc test based on standardized residuals), and Fisher’s exact test was used when some of the categories were small. The normality of the distributions was verified with the Kolmogorov-Smirnov test and through histogram analysis. The *p* = 0.01 was considered as the level of significance in the study. The reason for choosing such a level of significance is the small group of patients in some of the compared groups. A lower level of significance reduces the probability of considering a random result as statistically significant.

## Results

A total of 319 adult patients (178 women and 141 men) with confirmed CDI were enrolled in the study. 80 people died (50 women and 30 men). The mean age of the patients was 72.08 (SD = 16.74) years. Data on the number of hospitalizations, morbidity and mortality are presented in Table 1.

**Table 1:** Analysis of mortality in Clostridioides difficile infection and overall mortality at the University Clinical Hospital in Wroclaw, Poland, 2016–2018

Year of hospitalization	2016	2017	2018	2016–2018
Number of hospitalizations *	51,107	53,482	79,140	183,729
Number of patients with CDI	79	118	122	319
Percentage of hospitalizations with CDI	0.15%	0.22%	0.15%	0.17%
Morbidity (per 100,000 hospitalizations)	155	221	154	174
Number of deaths*	851	860	1089	2800
Number of deaths of patients with CDI	20	31	29	80
Percentage of deaths of patients with CDI	2.35%	3.60%	2.67%	2.86%
Overall mortality rate*	1.67%	1.61%	1.38%	1.52%
Mortality rate of patients with CDI	25.32%	26.27%	23.77%	25.08%

*Note* * Excluding pediatric wards and the Hospital Emergency Department

*Abbreviations* CDI, Clostridioides difficile infection

Over the entire period studied, the morbidity was 174 cases per 100,000 hospitalizations. Mortality in the study period was high and amounted to 25.08%. It exceeded more than 15 times the overall mortality rate among all hospitalized patients (excluding pediatric and emergency wards).

Statistical analysis of selected quantitative and qualitative variables in the context of deaths of patients with CDI was performed. The group of deceased patients with CDI exhibited several distinctive characteristics compared to survivors. Deceased patients were, on average, 9.24 years older and had a significantly longer duration of hospitalization, with a median length extended by 10 days. The analysis revealed lower albumin levels (Rho = -0.235, *p* < 0.001), higher urea levels, increased use of antibiotics, and a higher risk of malnutrition, as indicated by the NRS (Rho 0.219, *p* < 0.001). Furthermore, the deceased cohort demonstrated a higher incidence of comorbidities such as sepsis, heart failure, stroke, and hypothyroidism. Pneumonia was diagnosed twice as frequently among the deceased patients. Notably, the study highlighted a significant association between mortality and the use of specific antibiotics, with deceased patients more likely to have taken penicillin and fluoroquinolones. These findings underscore the importance of considering age, hospitalization duration, nutritional status, comorbidities, and antibiotic usage patterns in identifying individuals at a higher risk of mortality in the context of CDI. Detailed clinical characteristics of the patients are included in Tables 2 and 3.

**Table 2:** Analysis of selected quantitative variables of patients hospitalized with Clostridioides difficile infection at the University Clinical Hospital in Wroclaw, Poland, in 2016–2018 depending on patient survival

Variable	The patient survived	The patient died	*p*
Number of patients	239	80	
Age [years] (mean (SD))	69.76 (17.10)	79.00 (13.54)	< 0.001
Length of hospitalization [days] (median [IQR])	22.00 [21.5]	32.00 [37]	0.008
Erythrocyte sedimentation rate [mm/h] (median [IQR])	36.00 [38]	32.00 [22.25]	0.722
C-reactive protein [mg/l] (median [IQR])	52.44 [116.24]	87.04 [124.9]	0.047
Procalcitonin [ng/ml] (median [IQR])	0.23 [0.54]	0.27 [0.76 = 0.89 − 0.13]	0.341
Leukocytes [thousands/µl] (median [IQR])	9.70 [6.61]	10.09 [6.61]	0.345
Erythrocytes, female [million/µl] (mean (SD))	3.85 (0.68)	3.84 (0.81)	0.929
Erythrocytes, male [million/µl] (mean (SD))	4.02 (0.82)	3.99 (0.93)	0.880
Hematocrit, female [%] (mean (SD))	34.41 (5.98)	34.82 (6.66)	0.707
Hematocrit, men [%] (mean (SD))	36.26 (7.02)	36.26 (7.91)	0.998
Hemoglobin, Female [g/dl] (mean (SD))	11.24 (1.96)	11.35 (2.15)	0.758
Hemoglobin, male [g/dl] (mean (SD))	12.02 (2.49)	11.81 (2.52)	0.684
Thrombocytes [thousands/µl] (median [IQR])	236.50 [145.25]	241.50 [170]	0.560
Sodium [mmol/L] (mean (SD))	137.71 (5.48)	139.04 (6.31)	0.073
Potassium [mmol/L] (mean (SD))	4.07 (0.70)	4.20 (0.83)	0.200
Magnesium [mg/dl] (mean (SD))	1.93 (0.40)	2.02 (0.51)	0.141
Calcium [mg/dl] (mean (SD))	8.94 (0.80)	8.98 (1.34)	0.827
Total protein [g/dl] (mean (SD))	5.86 (0.93)	5.61 (1.28)	0.119
Albumin [g/dl] (mean (SD))	2.95 (0.68)	2.61 (0.81)	0.002
Creatinine [mg/dl] (median [IQR])	1.06 [0.74]	1.14 [1.32]	0.062
Urea [mg/dl] (median [IQR])	41 [40]	53.50 [50.25]	< 0.001
Number of antibiotics (median [IQR])	2.00 [2.00]	3.00 [2.00]	< 0.001
Nutritional Risk Score (mean (SD))	1.79 (1.35)	2.68 (1.39)	< 0.001

*Abbreviations* SD, standard deviation; IQR, interquartile range

**Table 3:** Analysis of selected qualitative variables of patients hospitalized with CDI at the University Clinical Hospital in Wrocław in 2016–2018 depending on the patient’s survival

Variable	The patient survived	The patient died	*p*
Number of patients	239	80	
Sex = Female (%)	128 (53.6)	50 (62.5)	0.206
Year of hospitalization (%)			0.904
2016	59 (24.7)	20 (25.0)	
2017	87 (36.4)	31 (38.8)	
2018	93 (38.9)	29 (36.2)	
Patients admitted with diarrhea = Yes (%)	46 (19.2)	13 (16.2)	0.666
Urinary tract infection = Yes (%)	91 (38.1)	33 (41.2)	0.710
Pneumonia = Yes (%)	53 (22.2)	36 (45.0)	< 0.001
Sepsis = Yes (%)	26 (10.9)	21 (26.2)	0.001
Stroke = Yes (%)	44 (18.4)	27 (33.8)	0.007
Dementia = Yes (%)	37 (15.5)	23 (28.7)	0.014
Hypertension = Yes (%)	135 (56.5)	49 (61.3)	0.538
Ischemic heart disease = Yes (%)	58 (24.3)	24 (30.0)	0.386
Myocardial infarction = Yes (%)	30 (12.6)	19 (23.8)	0.026
Heart failure = Yes (%)	42 (17.6)	40 (50.0)	< 0.001
Atrial Fibrillation = Yes (%)	68 (28.5)	33 (41.2)	0.046
Diabetes = Yes (%)	70 (29.3)	19 (23.8)	0.417
Renal failure = Yes (%)	71 (29.7)	36 (45.0)	0.018
Crohn’s disease or ulcerative colitis = Yes (%)	13 (5.4)	0 (0.0)	0.044
Pressure ulcer = Yes (%)	29 (12.3)	10 (12.5)	1.000
Surgery = Yes (%)	32 (13.4)	11 (13.8)	1.000
Hypothyroidism = Yes (%)	15 (6.3)	14 (17.5)	0.005
Anemia = Yes (%)	161 (67.4)	61 (76.2)	0.175
Active oncological process = Yes (%)	38 (15.9)	19 (23.8)	0.156
Use of proton pump blockers = Yes (%)	139 (58.2)	45 (56.2)	0.866
Use of ranitidine = Yes (%)	28 (11.7)	6 (7.5)	0.402
Use of penicillins = Yes (%)	77 (32.2)	45 (56.2)	< 0.001
Use of cephalosporins = Yes (%)	123 (51.5)	47 (58.8)	0.317
Use of carbapenems = Yes (%)	55 (23.0)	26 (32.5)	0.124
Use of fluoroquinolones = Yes (%)	92 (38.5)	45 (56.2)	0.008
Use of aminoglycosides = Yes (%)	26 (10.9)	14 (17.5)	0.176
Use of macrolides = Yes (%)	11 (4.6)	6 (7.5)	0.387
Use of sulfamethoxazole and trimethoprim = Yes (%)	20 (8.4)	8 (10.0)	0.651
Use of colistin = Yes (%)	20 (8.4)	10 (12.5)	0.382

## Discussion

Morbidity of CDI in the world and in Poland differ significantly. This is related, among other things, to the different characteristics of the health care system in each country and the different structure of hospitals. The occurrence of new strains also affects the morbidity, such as the NAP1 strain – North American Pulsed Field Type 1, which was the cause of the increase in the morbidity of CDI in the USA in 2005 [20]. At the University Clinical Hospital in Wroclaw, in Poland, the morbidity of CDI in the adult population in 2016–2018 was 174 cases per 100,000 hospitalizations and was lower than hospitals of a similar profile in Poland [15, 21].

Similarly, a wide range of CDI mortality in different centers around the world is observed, ranging from 9 to 38%, depending of literature [13, 14]. In our study, CDI is characterized by a high mortality rate, which was 25.08% and exceeded the overall hospital mortality rate 16 times, and was much higher than in other hospitals in Poland with a similar profile [15]. Therefore, it is worth looking into the factors that may contribute to such a high mortality rate.

In our study, we showed that people suffering from CDI were characterized by: older age, multimorbidity, long hospitalization period, use of a broad-spectrum antibiotic (cephalosporin, fluoroquinolone, penicillin) during or before hospitalization. The mean age of the patients was 72.08 years and 72.73% of them were over 65 years of age. Our results are similar to those of the study by Zilberberg et al. [22] conducted in the United States in 2000–2005 and the results of the British report from 2004 [23]. In a similar facility in Poland, the average age of patients and the percentage of patients over 65 were lower [15], which may at least partly explain the lower mortality rate in this hospital.

Advanced patient age is also a risk factor for death in CDI, as documented in many studies [24–28]. In our study, mortality increased with patient age, and those who died were on average 9.24 years older than patients who survived CDI. It is worth citing the meta-analysis by Bloomfield et al. [29], in which older age, as well as increased white blood cell counts, elevated creatinine levels, and use of corticosteroids were associated with increased mortality in CDI.

Another risk factor is the length of hospitalization, which in our study was 10 days longer for patients who died in our study. An analogous conclusion was reached by Dudukgian et al. [30], who analyzed the cases of 398 patients treated between 1999 and 2006 and observed increased mortality in patients who were hospitalized longer before CDI was detected.

In the group of deceased patients, we observed the presence of hypoalbuminemia, which is a typical disorder in the course of CDI, and elevated urea levels, as a sign of impaired renal function and dehydration. These parameters may have prognostic value in the context of patient death. Similar results were presented by the authors of a systematic review [25], a retrospective observational study [31], a cohort study [32] and other studies [33, 34]. Kikuchi et al. [35] showed a relationship with higher 30-day mortality in patients with a higher leukocyte count, higher urea, creatinine and lowered albumin concentrations in patients with CDI treated in the department of internal medicine at Shirikaba-Dai Hospital in Sapporo in Japan between 2010 and 2018.

We also showed that a higher rate of death was associated with a higher NRS, i.e., a higher risk of complications related to nutritional status. The NRS is rarely used to assess mortality in CDI. The influence of malnutrition on the course of the disease is more often described [27, 36]. In the study by Kyne et al. [37], the authors, in addition to malnutrition, also indicate the influence of other factors, such as: dehydration, fecal incontinence, physical disability, cognitive impairment, and recent endoscopy of the gastrointestinal tract.

We also showed that the patients who died had a higher incidence of pneumonia, sepsis, heart failure, stroke (whether hospitalized or in history), and hypothyroidism. Our results are similar to those of other authors. In a multi-center study by Cózar et al. [5], mortality among patients with three aggravating factors, such as heart failure, kidney failure, cancer, diabetes was twice as high as in patients with two factors. Particularly noteworthy are cardiovascular diseases, the presence of which increases the risk of death [25], especially the presence of ischemic heart disease [33] or heart failure [38], in which case mortality increased to approx. 50% in our study.

In our study, patients with a stroke during hospitalization or a history of stroke were more likely to die. The impact of damage to the central nervous system and disorders of its functioning on the course of CDI is rarely discussed in the literature. In the few works dealing with this subject: Appaneal et al. [27] and Bishara et al. [39] included cognitive impairment among the risk factors for death in the course of CDI.

We did not find any studies confirming the relationship between hypothyroidism and the death of patients with CDI in the analyzed literature. It seems, however, that the slowing down the peristalsis of digestive tract and the weakening of gastric acid secretion, which may occur in hypothyroidism, underlie the more severe course of CDI [40].

Pneumonia is an independent risk factor for death during hospitalization, which has been demonstrated, among others, in the meta-analyses of Loke et al. [41] and Viasus et al. [42]. It is also worth noting that broad-spectrum antibiotics are used to treat pneumonia, which increases the risk of CDI. In our study, pneumonia is a risk factor for increased mortality in the course of CDI.

Additionally, it is pertinent to mention that, consistent with our findings, Redelings et al. [26] identified sepsis as a contributing factor to increased mortality. This underscores the broader impact of severe infections and associated complications on the outcomes of patients with CDI.

Despite extensive studies, it seems that the assessment of the impact of specific disease entities on the course of CDI and the risk of death requires further research.

As in other studies [27, 31], in our study deceased patients received significantly more antibiotics during hospitalization. We showed that these were penicillin and fluoroquinolone antibiotics. Similar studies also indicated other beta-lactams and clindamycin [31].

## Conclusions

Our study at the University Clinical Hospital in Wrocław from 2016 to 2018 revealed a lower incidence of CDI compared to similar hospitals in Poland, yet a higher mortality rate. Advanced age, prolonged hospitalization, lower albumin, higher urea, malnutrition, and comorbidities like heart failure, stroke, pneumonia, sepsis, and hypothyroidism were associated with increased mortality. Furthermore, the heightened use of antibiotics, particularly penicillin and fluoroquinolones, correlated with elevated mortality risk. These findings emphasize the need for tailored interventions to mitigate CDI-associated mortality, particularly among high-risk patients.

## Data Availability

The datasets generated and/or analyzed during the present study are available from the corresponding author (PP) upon reasonable request.

## Abbreviations

CDI: Clostridioides difficile infection
NRS: Nutritional Risk Screening
